# Monographie des Dégenérations Skirrheuses de l'Estomac, fondée sur un grand nombre d'Observations recueillies tant à la Clinique de l'Ecole de Médecine de Paris, qu'a l'Hôpital Cochin

**Published:** 1817-09

**Authors:** 


### PART II.

### COMPREHENSIVE ANALYTICAL REVIEW OF MEDICAL LITERATURE.

"Tros, tyriusve, nobis nullo discrimine agetur."

*Monographie des Dégenérations Skirrheuses de l'Estomac, fondée sur un grand nombre d'Observations recueillies tant à la Clinique de l'Ecole de Médecine de Paris, qu'a l'Hôpital Cochin.* Par Frederic Chardel, D. M. Médecin de l'Hôpital Cochin, &c. 8vo. pp. 216. A Paris.

This excellent "*Monograph on scirrhous Affections of the Stomach*" is the production of Dr. Chardel, a disciple of the celebrated Corvisart, to whom the volume is inscribed.

Although a publication of no very recent date, we feel persuaded that, in announcing it, we shall introduce to the acquaintance of the general practitioner a work, the contents and even title of which are little known within his sphere of reading and conversation; and we are incited to the labour of its analysis by the hope of conferring no mean benefit upon those to whom the original is inaccessible, but who prefer the researches of the dead-house to the abstract and commonly futile speculations of the closet, and regard a correct knowledge of the anatomical character and varieties of a disease quite as essential to sound nosological arrangement and successful practice, as vigilant observation of the external phænomena which it presents. To such, then, our analytical sketch is dedicated: and may the ardour displayed by the enlightened foreigner in the prosecution of his pathological inquiries, exert a benignant influence upon those for whom we write, and arouse them to emulate his example. For never, be it remembered, is the physician or surgeon more honourably and usefully occupied than in tracing the connection between the seats and symptoms of diseases, and thus wringing from the dregs of human suffering and infirmity their most effectual antidote or balm.

Dr. Chardel, in his *Preface,* makes some observations on the predisposition of the various organs to organic disease. This is greatly influenced by sex, age, hereditary peculiarities of constitution, and the operation of other physical and moral causes. Thus, more than thirty persons, who perished from tubercular phthisis at the hospital *Cochin,* in 1807, were almost exclusively females; while, on the contrary, women are much less frequently than men the victims of gastric lesion, and furnish but one-fourth of the cases recorded by Corvisart in his work on diseases of the heart.

Some viscera are more prone than others to organic lesion. The lungs, the heart, the stomach, and liver, occupy successively the highest points in Dr. Chardel's scale of morbid susceptibility; and, as a proof that organs of permanent action are not exclusively subject to this species of lesion, he contends that the brain, which presides over the functions of all the rest, rarely exhibits examples of it. Now, from a retrospect of our own experience in the observation of diseases, we are certainly inclined to believe that both the liver and brain suffer more frequently than the stomach, from structural lesion; and this inference will, we think, be amply confirmed by an appeal to the records of pathological research. Yet, with respect to the cerebral organ, candour requires that one source of frequent delusion should be cursorily exposed. A train of morbid phænomena, which the hasty or inexperienced observer has directly referred to an affection of the brain itself, is very commonly proved by the operation of medicine, or by dissection, to have been utterly unconnected with lesion of the encephalon; and merely sympathetic irritation propagated to it from some remote organ, the actual source of the disease. This is particularly liable to occur in young subjects, and those who suffer much from intestinal torpor and irregularity.

Deaths from organic lesion are, in relation to those which result from other causes, computed to take place in the proportion of three to four. Out of 146 cases of fatal disease, furnished by the hospital *Cochin*, in the year 1807, sixty belong to the former class, and eighty to the latter. Most chronic diseases, indeed, when not dependent on the nervous system, or the operation of some virus, proceed from visceral affections; and as lesions of structure are, when they have attained a certain point, commonly incurable, it becomes highly important to detect them in their origin: for thus only can we hope to cut short or retard their progress. Correct anatomical knowledge and great practical acuteness are required for the diagnosis of an incipient lesion.

From some anatomical remarks on the mode of developement of gastric scirrhus, communicated by M. Dejaer, we learn that the parietes of the stomach presented the following alterations in several scirrhi, which diminished as they spread from the pyloric towards the cardiac orifice. The three membranes of the viscus were first separated by dissection, commencing at the sound portion. The mucous membrane became thickened, and adherent to the muscular; and the cellular structure, which connects the latter with the serous, was diseased, and separated the fibres of the muscular coat, but they remained long visible. The serous coat, yet preserving exteriorly its characteristic polish and lustre, was then intimately blended with the morbid cellular structure. On approaching the pylorus, the stomach acquired an increased thickness, chiefly resulting from tumefaction of the cellular structure. The fibres of the muscular coat still remained distinct, but widely separated and much paler than natural, far beyond the point where separation of the gastric membranes was longer impracticable.

Lastly, there was ulceration of the mucous membrane. Hence it appears, that the morbid action is propagated from the mucous membrane to the contiguous cellular structure, which becomes the principal seat of the affection; and that the muscular and serous membranes are only secondarily affected.

The *Historical notice* which succeeds, needs not detain us here. It consists of allusions to the writings of Hippocrates,* Galen,† Hildanus, Rivière, Storck, Lieutaud, and Haller, on the subject of lesions of the stomach; and concludes with the citation from Morgagni, of three cases of enlarged pancreas, in which vomiting constituted the principal phænomenon.

Dr. Chardel proposes to adopt in the composition of his work the method of induction pointed out by our immortal Bacon; and hopes, from a number of facts rigorously selected and exposed, and cautiously examined in all their bearings, to deduce some truths useful in themselves, and which may eventually lead to new discoveries. In conformity with this plan, the three first sections of the monograph constitute the principal exposition of facts; they are exclusively devoted to the history and morbid anatomical description of numerous cases of scirrhus, as affecting the cardia, the body, and the pyloric portion of the stomach. The causes, the signs and characters, the diagnosis and treatment of scirrhous affections of this organ, are then successively reviewed; but the three sections of pathological record are, we calculate, all that our time and limits will allow us to include under our present article.

Section the First.—*Scirrhous Affections of the Cardia.* Five cases of this variety of gastric lesion are here detailed. We shall select for transcription that which

---

* Dr. Chardal here takes occasion to remark, that the disease described by Hippocrates, under the title of μελαινα, "displays some resemblance to scirrhus of the stomach." On referring to the original description, we find ourselves utterly incapable of recognizing any such analogy between the two affections. *Melaina* is the *morbus niger* of modern writers. See that article in the *London Medical Dictionary*, vol. ii.—Useful information respecting the pathology of this singular affection may also be acquired from Dr. Home's *Clinical Experiments, Histories, and Dissections*, page 120-136.

† This ancient writer has observed, that there sometimes arises in the stomach a wart, or fleshy tumour, by which the passage of the aliment may be impeded or even obstructed.

appears to us to be most interesting and minutely recorded: it constitutes the second in Dr. Chardel's series.

*Case.* A lady, of Lyons, aged 33, of a robust but apparently delicate constitution, little subject to disease, although constantly complaining, had some years previously, suffered repeated attacks of colic. In June 1777, this affection was renewed, and the symptoms then were fever; clammy and bitter mouth; vomiting of greenish matter; pain, alternately acute and obtuse, in the umbilical and hypogastric regions, and aggravated by pressure; distension of the abdomen; skin dry and parched; and urgent thirst. There was neither pain nor sense of weight in the stomach. By the employment of blood-letting, an emetic, diluents, sedatives, emollient cataplasms to the abdomen, and baths, she recovered in a fortnight; but from this period, she frequently complained of an indefinite sense of uneasiness in the stomach.—On the 23d of February, 1779, the patient again required medical attendance. About three weeks previously, she had been seized with fever, vomiting, pain in the stomach and abdomen, and repeatedly taken purgative medicines. She was now without fever, but had frequent vomitings, with scarcely any effort, of a deep yellow matter. The alvine discharges were rare, difficult, and scanty; the abdomen distended and very painful, chiefly towards the right hypogastrium; and the epigastric region tense without induration. The abdominal pain was increased by pressure. Wakefulness, loss of appetite; continual agitation and inquietude; bitterness and dryness of the mouth, with hard pulse, were the other symptoms. The vomiting became insensibly more frequent. Fluids, ere they could reach the stomach, were rejected without effort. After some days, they were of a brown colour, but without smell; the emaciation increased. The stools became more infrequent, liquid, and nearly of the same colour as the fluid ejected by vomiting. Acute fever, on March 2d, was added to the preceding symptoms. The abdominal distension increased, and the pains became keen and lancinating; the pulse very hard; the skin hot and dry. Hiccough and strangury ensued. It was now thought that a great portion of the intestinal canal was scirrhous, but that the stomach and pylorus were perfectly sound; for the vomiting of fluids succeeded their ingestion, ere they apparently could have passed the œsophagus. That the entrance of fluids was opposed by the compression which the tense and obstructed intestines exercised upon the gastric organ, seemed to be the most probable conjecture.

*Dissection.* About eight pints of a yellowish and extremely offensive fluid were effused into the cavity of the abdomen. The pancreas was scirrhous; the liver appeared to be perfectly sound, and of the wonted volume; but its substance was exceedingly soft and crumbly, yielded readily to the impression of the finger, and became reduced to a soft and grained paste. The gall-bladder, kidnies, and spleen, were healthy; but the latter unusually small. The œsophagus was horn-like, and completely scirrhous in its whole circumference at its union with the stomach, and one finger's breadth above; in this portion, the tube was nearly obliterated: a clear yellow fluid was contained in it. The superior orifice of the stomach was absolutely scirrhous throughout; and there were "scirrhous concretions" to the extent of two fingers' breadth around it. The stomach was empty but very flaccid, much shrunk, and, as it were, crushed by the pressure of the intestines. The pylorus was quite sound. The small intestines, were scirrhous nearly in their whole length, their parietes thickened, and presenting a horn-like induration, and their cavity much contracted. The large intestines, inflamed through the greater part of their track, exhibited some black and gangrenous spots. There was no air in the abdominal cavity, or in the intestines.

The four remaining cases we can only allow ourselves to sketch with the utmost possible brevity. *Case* 1. *Sex,* male; *age,* 38; *temperament,* melancholic; *general health* precarious; had been some years subject to violent colic and stomach-ache.—*Symptoms.* Dysphagia for more than a year; painful vomiting of aliment and often mucus: nothing discoverable by the touch; deglutition subsequently relieved, but frequent vomiting; obstinate constipation; relapse with violent pains of the stomach; countenance now pale, yellow, earthy; appearance cachectic; constant heavy epigastric pains; pulse frequent; constipation unsubdued. Again relieved, but vegetable substances only could be retained; constipation and cachectic expression continued. Speedily relapsed; unable to speak from exhaustion; pointed to the throat as the seat of his distress; deglutition nearly impracticable; extreme depression; extremities cold; pulse imperceptible; complete aphonia; death.—*Morbid appearances.* A cyst, two inches deep and one and a half in diameter, containing three spoonfuls of pus, beneath the left clavicle. The œsophagus sound in the part corresponding to the cyst, but much thickened and affected with an ulcerated scirrhus, implicating the cardiac orifice, and extending four inches above. The bodies of the dorsal vertebræ opposite the scirrhous portion of the œsophagus softened so as to yield readily to the scalpel. The intervertebral ligaments nearly putrid.

*Case* 3. A male, aged 50, of strong constitution and regular habits, had been for some years declining.—*Symptoms.* Foul mouth; gradual loss of strength and appetite; stomach greatly disordered; pains at the heart; nausea; constant spitting for four months past. Mouth worse; frequent vomiting, particularly after meals, of a glairy saliva without aliment; hence dread of eating. The fluid ejected very abundant, ropy, viscous, acid, highly disagreeable to the mouth. Debility extreme; inferior extremities, abdomen, and face, puffy. The mouth dreadfully foul and offensive. All the pain referred below the ensiform cartilage, and a small tumour obscurely felt by pressure there. Constipation now habitual; frequent colics; urine irregular; deglutition, even of fluids, difficult. The spitting and vomiting for a time relieved by sedatives, and the mouth less offensive, but the gastric pain and constipation continued: pulse weak and low. Death took place without a struggle: on the day preceding it the vomiting had re-commenced.

*Dissection.* An ulcerated scirrhus, extending from the cardiac orifice and commencement of the œsophagus to the smaller arch of the stomach; a groupe of scirrhous glands composing the summit of the tumour; which, towards the cavity of the organ, had the aspect of the syphilitic cauliflower excrescence, and formed a frightful ulcer, of an acid and intolerably offensive smell. Above, it adhered intimately to the gastro-hepatic (little) omentum, the convex surface of the middle hepatic lobe, and to the pancreas, which was healthy. But the liver, at the point of adhesion, displayed a large tubercle, white, and resembling *adipocere.* The pyloric orifice perfectly sound.

*Case* 4. A widow, aged 64. *Symptoms.* Epigastric and lumbar pains; indigestion; constipation; vomiting of half digested acid food, mixed with an albumen-like substance. Solid aliment at length rejected; warm liquids alone retained; vomiting very frequent; loss of flesh and strength; much suffering from obstructed flatulence; constipation removed by coffee; evacuations yellow or blackish: a violent cold, succeeded by swelling of the legs. An emaciated and worn appearance; tongue clear; breast only painful during the cough, by which a copious puriform fluid was expectorated; abdomen swollen; and between the right (left?) hypochondrium and epigastrium, a large irregular tumour obscurely felt, firm, and painful on rude pressure. The body in the first stage of atrophy; painless vomiting and diarrhœa; swelling of the left arm, and utter exhaustion, preceded the fatal issue.

*Dissection.* Twelve pints of dark red fluid effused into the abdomen: The liver pale, hard, and scirrhous, adhering towards the left near the suspensory ligament, to the stomach: The gall-bladder wanting; a deep round cicatrix marking its wonted site: The omenta indurated, shrunk, and inseparably adherent to the stomach, dividing that organ into two reservoirs which communicated together: The pancreas compact and scirrhous; the spleen shrunk, and both forming by their morbid connections with the stomach an indiscriminate mass: The cardiac orifice hard, scirrhous, thickened, contracted; and the gastric extremity of the œsophagus narrowed and ulcerated to the distance of an inch above the diaphragm: The cardiac portion of the stomach (which constituted one of the reservoirs) hard, thickened, ulcerated, beset with fungous excrescences, and so contracted as scarcely to hold a hen's egg: The other portion of the stomach and the pylorus sound.

*Case 5.* A male, aged 62, worn down by protracted quartan fever.—*Symptoms.* Vomiting, for fifteen months past, near meal-time; no constipation; stools pitchy and tenacious; nothing particular found on external examination; extreme marasmus preceding death.

*Dissection.* The parietes of the stomach flaccid, and contracted in its centre; but apparently sound in its exterior: The superior border of the organ adhering so strongly to the concave surface of the left lobe of the liver, that the former was lacerated in the separation, and thus a large ulcer of the liver exposed, which had spread to the diaphragm and corresponding portion of the pericardium, the latter having suppurated: The inferior surface of the stomach confounded with the inferior of the spleen: The interior of the cardiac orifice completely surrounded by a scirrhus, yielding a blackish and intolerably offensive sanies; and pus inclosed in the substance of the gastric parietes.

Thus, it appears that of the *five cases* of scirrhus implicating the *cardiac orifice* of the stomach, which are here recorded, *three* occurred in the *male*, and *two* in the *female* subject; and *all* in the *middle* or *decline* of life, that is, from about the thirty-fourth to the sixty-fifth year. *Vomiting*, commonly *soon after* the ingestion of food, and *extreme emaciation* were present in *all* the cases, and obstinate *constipation* in *four* of them: in *two* of them, a *hard tumour* was felt on examination of the epigastric region; but in *one* only were there *dysphagia*, and *loss of voice*. *Four* of the cases were complicated with considerable lesions of the other abdominal organs, principally the *liver*, *spleen*, and *pancreas*; *two* with *peritonæal dropsy*, *one* with *scirrhus of the small intestines*; and, in *one*, the *dorsal portion of the vertebral column* was diseased.

Section the Second. *Scirrhous Affections of the Body of the Stomach.* We shall here, as in the preceding section, present an extended and nearly literal translation of one of Dr. Chardel's histories, and merely trace a general outline of the rest. They are five in number. We select the fourth.

*Case.* A joiner, aged 47, addicted to morning potations of brandy, had been drooping for a year. A diarrhœa, with which he had been at first seized, was suddenly suppressed by medicine. A fixed pain in the right hypochondrium succeeded; and the alvine evacuations soon again became frequent. Various emollients and discutients were unavailingly applied to the seat of pain. The man gave up his work; and lost his flesh and appetite. The pains were aggravated. The stools became blackish, and the lower limbs œdematous. This was his condition on entering the hospital. He had never been ill before. Acid eructations now distressed him. The right hypochondrium was tense, and constantly painful, particularly on pressure of the corresponding inferior region of the chest, or of the soft parts between the three first false ribs near their cartilaginous portions. There was no vomiting. The pains were greatly soothed by the application of poultices, and internal employment of opium. Yet the stools became more black and abundant, and frequently mixed with blood. The catechu was tried in vain. Dropsy and marasmus increased: and the patient sank exhausted.

*Dissection.* Twenty-four hours after death, the lower part of the thorax, corresponding to the seat of pain, displayed a livid greenish hue. The abdominal cavity contained a considerable quantity of pure serum. The gastro-colic (great) omentum, indurated and folded back upon the stomach, adhered to that organ, the diaphragm and liver.

The centre of the arch of the colon was united to the great curvature of the stomach; and the calibre of the intestine was, at this point, diminished. The centre of the great curvature of the stomach had contracted strong adhesions to the diaphragm. These destroyed, a small scirrhous ulcer was discovered, penetrating more towards the interior, than spreading on the external surface of the stomach, the morbid paries of which was at least two centimetres (more than two thirds of an inch) in thickness. A blackish sanies coated it. The diaphragm, indurated and scirrhous at the point of adhesion, on its abdominal surface only, supplied the place of that portion of stomach which had been corroded by the ulcer. The gastric organ, in other respects, was sound: but both it and the whole track of the intestinal tube, contained part of the sanies which had escaped from the ulcer. The liver was enlarged, and so much indurated that it cracked beneath the scalpel. The gall-bladder, shaped like an intestine, was filled with discoloured bile.

*Case* 1. A colour-grinder, *age* about 55, *temperament* bilious. *Symptoms:* Sense of pain and weight in the stomach after eating, with vomiting of the food; obstinate costiveness: no painters colic. After death, a considerable depression observed below the ensiform cartilage.

*Dissection.* A large but not ulcerated scirrhus, apparently formed of coagulated lymph, extending from the pylorus exclusively to the lesser arch of the stomach, which was nearly effaced. The lymphatic glands of this region forming a sort of papillary tumour. The internal membrane of the great arch presenting numerous rugæ: the small intestines in several points narrowed. The neck of the gall-bladder contracted, and its situation altered by intimate adhesion to the pylorus.

*Case* 2. A male, age 57, delicate, long exposed to mental suffering. *Symptoms:* Indigestion following a cold, and obstinate dry cough; sense of fulness and constant uneasiness in the stomach; bowels torpid: great disturbance and severe pain after food, aggravated by purgatives, opium, and warm bath; debility; broken sleep. Appetite unimpaired but sufferings dreadfully aggravated by taking food; nausea from time to time and vomiting. A hard substance, painful on pressure, obscurely felt in the epigastric region.

*Dissection.* A scirrhous tumour, without ulceration, formed by tumefaction of the superior parietes of the stomach and of the lymphatic glands of the little omentum, extending along the smaller curvature of the stomach: Its internal surface simply corrugated; the pancreas compact, enlarged, and presenting an embossed appearance. The concave surface of the middle lobe of the liver variegated by white, hard, fatty tubercles.

*Case* 3. A widow, age 38, healthy till within the last six years, and a dreadful sufferer in the storms of the revolution, attacked successively by uterine hæmorrhage, jaundice, and peripneumony; and, during convalescence from the latter, with pains at the stomach, nausea, and vomiting on ingestion of food, solid or liquid. A very large tumour in the left of the epigastric region; pains diffused all over the abdomen, with violent diarrhœa; weakness; extreme emaciation. Mouth unpleasant; tongue clean; urgent thirst; the epigastric tumour projecting, spherical, painful; abdomen generally sore; the stomach incapable of bearing solids or liquids, the former even swallowed with difficulty; continued diarrhœa. Before death, vomiting less frequent, and ejected fluid, of a fæcal odour.

*Dissection.* The large omentum adherent to the peritonæum of the pubic region: Both curvatures of the stomach forming a large tumour, in which the adjacent lymphatic glands were confounded. A foul and offensive ulcer on the tumour towards the cavity of the organ. The cardia and pylorus sound. Numerous, middle-sized, whitish, hard tubercles in the substance of the liver. The internal membrane of the colon corroded by small sanious ulcers: the external aspect of the intestine healthy.

*Case* 5. A male, age 49, delicate but heretofore healthy, and addicted to irregular habits of alternate drinking and abstinence. *Symptoms*: Diarrhœa, pains at the stomach, and occasional vomiting, first about the 40th year, but subsequently aggravated; weakness; extreme emaciation. Vomiting frequent; diarrhœa alternating with obstinate constipation. The epigastric region occupied by a large, hard, circumscribed, indolent tumour. Vomiting now excited by the slightest aliment, sometimes hard and painful, sometimes without effort; stools blackish; anasarca; frightful emaciation; death.

*Dissection.* Abdominal effusion of serum; the lesser curvature of the stomach, near the pylorus, occupied by a large oblong scirrhus, on the verge of ulceration; the long diameter of the tumour extending, not in the direction of the small curvature, but from the posterior to the anterior surface of the stomach: The anterior extremity of the scirrhus terminating in a sort of *cul-de-sac,* much resembling one of the gastric orifices. A portion of the liver confounded with the scirrhus; the pylorus quite sound, but with its valve effaced, situated about the centre of the right boundary of the scirrhus; and concealed beneath a transverse membranous production of the villous coat, with which its extremities were confounded: On the left border of the scirrhus, a considerable perforation.

Upon a retrospect of these *five cases* of *central scirrhus*, we, then, find that the subject of *one* only was a *female;* that *two* of the patients had suffered from *deep-rooted moral affections, two* from *intemperance;* and that *all* were between the *thirty-second* and *fifty-fifth* year. *Pain in the epigastric region* was present in *all* the cases; *vomiting* in *four; extreme emaciation* in *three; constipation* in *two, diarrhœa* in *two;* and in *one, both the states alternating:* An *epigastric tumour* was discoverable, during life, in *three.* The *liver* was *tuberculated* in *two,* and *enlarged* and *indurated* in *one,* of these cases. *Two* were complicated with *peritonæal* or *general dropsy; two* with *ulceration* or *constriction of the colon: one,* with *contraction* of the *small intestine; one* with *enlarged pancreas;* and *one,* with *indurated omentum,* and *scirrhous diaphragm.*

Section the Third. *Scirrhus of the Pylorus.* Ten cases are here recited by Dr. Chardel. The first and fifth, as being most minutely detailed, we shall correctly transcribe; and exhibit the outline of the other eight in our wonted style of analytical brevity and compression.

*Case.* A man, aged 63, of sanguineo-lymphatic temperament, whose father had been healthy, but whose mother died, in middle age, of an evident affection of the stomach, had, notwithstanding severe mental distress, been always free from disease. Reduced by poverty to sleep in his clothes, on a damp floor, and overwhelmed with accumulated misfortune, he was attacked, in the summer of 1801, with anorexia, oppression in the epigastric and hypochondriac regions, palpitations of the heart, and costiveness. Ingestion of food, and *reclination on the left side,* aggravated his complaints. Till the middle of October, however, he continued his occupations; but was compelled, from the state of his stomach, to retrict himself to the allowance of eight ounces of bread daily, and a little light soup and brandy in the evening. Weakness and extreme emaciation shortly succeeded. The difficulty of going to stool gradually increased. He frequently rejected part of his food in a quarter of an hour after its ingestion, especially towards night. Oppression, a sense of weight in the epigastrium, pain in the left hypochondrium, and palpitations of the heart, were the consequences of its retention. Wine and sugar-candy passed more readily than any thing else. But the man's sufferings incessantly grew worse: and he entered the *Charité* in November.

A wasted figure, a ghastly countenance, and moist and glistening eyes deeply sunk in their hollow orbits were the prominent characters exhibited by the patient. But the skin was natural in colour; the pulse, infrequent, quick, irregular. The epigastrium was tense, and at its right inferior part was felt an oblong tumour extending from the cartilage of the false ribs nearly to the umbilicus. This tumour, commonly painless, was sensible to the slightest pressure; while the left hypochondrium, the usual seat of pain, admitted of examination without inconvenience. The hypogastric region shewed nothing peculiar, except a slight contraction of the muscles. The bowels were constipated; the urine clear and copious.

Food, taken towards night, was soon succeeded by a sense of constriction, anxiety, and weight in the lower part of the epigastrium, and general debility. This sensation seemed after to extend leftward to the hypochondrium, with severe shooting pains, and palpitations of the heart. In about half an hour, part of the food, mixed with phlegm, was ejected, and the remnant of the night was tranquil. Sedatives, given at night, seemed productive of relief. The vomiting ceased; and the patient thought himself better, and was able to lie on his back; when, one morning in January, he was found dead in bed. The puslse, for some days, had been small and low.

*Dissection.* The stomach, apparently sound in the exterior, was so large that its inferior third part was contained in the right hypochondrium. It was heavier than usual. A scirrhus of the mucous membrane almost wholly occupied its inferior third part, forming a kind of funnel or hollow cone, in the summit of which was the nearly obliterated pylorus. The scirrhus was compact in structure, yellow or dirty-white, irregular, rugous, and apparently of a fatty nature.

*Case.* A shoe-maker, aged 51, generally healthy, was admitted into the hospital, in July 1797. Some years before, he had suffered much from domestic troubles, and want of proper food. For about eighteen months, he had been harassed with nausea, unaccompanied by vomiting, although the fauces were artificially irritated to provoke it. Frequent fainting and lassitude rendered him incapable of his wonted exertion. Acute and fixed pains in the stomach succeeded. They were appeased by taking food, and re-appeared with violence proportioned to the time which had elapsed from the period of ingestion. In February, he vomited a very large lumbricus; and took, in consequence, a decoction of garlick in milk. Raw garlick, afterwards substituted for this, "set his bowels on fire," and left in his throat a sense of heat and constriction. The same acrid remedy, steeped in brandy, produced more violent effects; and the sense of constriction, now permanent, sometimes menaced suffocation. The bowels could only be unloaded by injections. Shortly afterwards, he was obliged to quit work, by acute and lancinating pains in the right hypochondrium, returning by frequent paroxysms, and complicated with a rending pain from the middle of the shoulders to the sacrum. In March, the abdomen rapidly enlarged, particularly on the right side. Swelling of the ankles came on; spread rapidly to the thighs; spontaneously disappeared, and returned in a permanent, but less active form. Clusters of cylindrical substances, supposed by the man to be putrid worms, were now voided by stool; and repeated emetics were administered without relief. The cylindrical substances again came away, in June.

On admission into the hospital, his countenance was pale, not yellow; his abdomen large and tense. An indurated substance, extending far both anteriorly and to the right, was felt beneath the abdominal parietes. This region gave out an obtuse sound; the left was softer. In the hypogastric, an obscure fluctuation was perceptible. The œdema scarcely rose above the knees: the appetite was good, but wine excited disgust. The nausea had disappeared; and the epigastric pains yielded, as soon as the abdominal swelling commenced, to a fixed tightness lower down in the same region, which was aggravated by taking food. Sleep was frequently broken by the pains of the back and right side: the urine natural. The œdema now rose rapidly to the thighs and scrotum: the abdomen grew larger; and the pains, become less acute, insensibly spread over the whole of it. A sensation of pricking recurred at intervals, particularly when the belly, otherwise not sore to the touch, was rudely handled. The constrictions of the throat became more frequent and intense; the skin was at times hot, and the pulse accelerated, especially towards evening; the urine decreased; the lips, tongue, and fauces, assumed a bright red colour; the tongue was unusually sensible, and its papillæ prominent. In July, the fluctuation was very evident in the lower half of the abdomen. The inferior extremities and perinæum were greatly distended, and the back and whole abdomen implicated in it: yet the indurated substance, in the higher region of the latter, could always be recognized. Dyspnœa increased; the pulse became small and frequent. Presently, the effusion extended to the chest, neck, and superior limbs; and a dew-like exudation of the fluid took place from the internal surface of the thigh, without evident breach of the epidermis. The man still regularly took his food, and had one motion daily: but, about the close of July, the dyspnœa was such as to interrupt his speech. Breathlessness, distress, and syncope, were induced by the slightest exertion. On the morning of the 26th, he took a little light food, and had a loose stool, of various colours. When placed in bed, he did not recover from the breathlessness consequent on motion. He pointed to the throat as the seat of suffocative feeling, and soon afterwards expired. This man had never been a drunkard, or subject to piles. He was confined to his bed but three days. His urine, though difficultly voided and scanty, had preserved its natural colour and limpidity.

*Dissection.* A large quantity of clear unaltered serum issued, upon incision, from the abdominal cavity. The liver was much enlarged; extending to the left hypochondrium, and occupying, anteriorly and to the right, nearly the superior half of the abdomen: it weighed eleven pounds. Large white tubercles, scattered through its substance, projected from all its surfaces. The largest equalled a hen's egg in volume. They appeared to be, particularly at their circumference, somewhat firmer than the parenchymatous structure, which retained its natural colour and consistence. The lesser extremity of the stomach, including the pylorus, was scirrhous to the length of three inches, and in its whole circumference, except towards the great curvature, where, for a finger's breadth, it appeared sound, even to the pylorus. It was six lines in thickness, and adhered strongly to the corresponding part of the liver. The interior of the scirrhous portion was rough and ulcerated: the duodenum and other intestines were apparently healthy; the colon enormously distended with gas. There was serous effusion into both cavities of the pleura, and into the pericardium. The heart was shrivelled: the surface of this organ, the lungs, the trachea, and œsophagus, were loaded with serum.

Nothing could be discovered in the throat, to explain the constriction of which the patient had so long and constantly complained; except that the larynx shared, in a high degree, the general effusion. The superior surface of the epiglottis was coated with a reddish gelatinous substance, at least three lines in thickness.

The following Table, which we have constructed for the purpose, will exhibit a brief outline of Dr. Chardel's eight remaining cases of scirrhous pylorus.

| | *Sex, Age, Habits, &c. of the Subject.* | *Morbid Phænomena observed during Life.* | *Morbid Appearances on Dissection.* |
|---|---|---|---|
| II. | Female, aged sixty-seven, commonly healthy.—Period from beginning of March to middle of April, 1800. | Spontaneous vomiting, preceded by epigastric pains, caused by mental affliction: fever; nothing discoverable externally; vomiting without effort, sometimes soon, sometimes many hours, after food; constipation; heat in the stomach, acidity; vomiting once subsided, but soon recurred; weakness; fatal hectic. | Corpse wasted to a skeleton; stomach so large, that the great arch extended to the ilia; pylorus pushed down towards the lumbar vertebræ, indurated, thickened, enlarged—its orifice a mere fissure, resembling the os tincæ; small intestines and cœcum pent up in the pelvis behind the uterus. |
| III. | Male, aged fifty-seven. Period from December, 1796, to November, 1797. | Epigastric pain, of four months' duration, often followed by vomiting; costiveness, emaciation; large tumour in the epigastrium; vomiting relieved, and even subsided for a time; other symptoms continuing; colics, debility, œdema, death. | Large but unulcerated scirrhus occupying the pylorus; and its orifice so much contracted as not to allow the passage of the smallest morsel of food. |
| IV. | Male, aged thirty-three, exposed to hard labour and hard fare; had suffered from sciatica, followed by pain, phlegmon, and suppuration in the right leg. Period from October, 1801, to February, 1802. | General uneasiness with shivering; frequent vomiting, bilious and spasmodic, relieved by medicine; oppression; epigastric pains; a hard and unequal tumour from the ensiform cartilage to the umbilicus; pains in the right hypochondrium and whole abdomen; evening fever, dyspnœa, disturbed sleep; face and epigastrium sweating with a generally dry and hot skin; depression, sinking pulse, violent delirium followed by temporary calm; convulsion of the facial muscles; thick offensive general perspiration, preceding death. | A hard and scirrhous tumour occupying the pylorus and vicinity of its valve, but not intercepting the communication between stomach and duodenum; glands of the lesser omentum remarkably tumefied; great omentum much wasted: pancreas scirrhous throughout; its constituent glandules much indurated without ulceration, and projecting from the surface; its investing peritonæal membrane slightly inflamed; gall-bladder distended with greenish bile; much greenish serum in the abdominal cavity; intestines largely inflated. |

| | *Sex, Age, Habits, &c. of the Subject.* | *Morbid Phænomena observed during Life.* | *Morbid Appearances on Dissection.* |
|---|---|---|---|
| VI. | Male, aged sixty-three, long the victim of mental distress. Period from middle of May to beginning of June, 1800. | Indigestion; stomach painful to the touch; bowels torpid; severe and lancinating epigastric pains; acidity; unpleasant eructations; fœtid breath; vomiting soon after meals, and the ejected matter stained by a blackish fluid. Costiveness obstinate; skin sallow and earthy; dreadful emaciation; death. | Stomach natural externally, not enlarged, but lodged in the left hypochondrium; pylorus removed from its wonted site, and thrice its natural volume, forming a hard cylindrical scirrhous mass, its orifice much contracted, and near it a round patch of the villous coat 4 inches diameter, red, hard, thickened, excoriated. Remains of vegetable food found in the stomach and duodenum. |
| VII. | Male, aged fifty, temperate, healthy; exempt from moral suffering. Period about 18 months, ending Dec. 1801. | Sense of weight in the epigastrium, followed, after 18 months, by purging and vomiting; digestion painful, food rejected in 5 or 6 hours after taking it, and the vomited matter stained by a soot like substance. Tongue clear, constipation obstinate; a hard indolent tumour between the epigastrium and umbilicus; figure so emaciated as to resemble a living skeleton; death. | A scirrhus tumour, larger to the left than right, extending from the lesser arch of the stomach, and implicating the pylorus; and forming, towards the stomach, an ulcerated fungus, apparently not the source of the black matter staining the interior of the organ. Pyloric orifice contracted; its valve thickened and indurated. The glands of the omenta, particularly those of the smaller, greatly tumefied. |
| VIII. | Female, aged sixty-six, and mother of several children. Period November to December, 1800. | Fixed pain, of three years' duration, in the epigastrium, growing progressively worse; vomiting of all the food; ejected fluid and breath sour; a hard tumour, painful on pressure, in this region, and extending to both hypochondria; an ulcer, repeatedly cicatrized, on the left leg. Habitual constipation; sense of constriction in the throat, embarrassing respiration in the erect posture; slight diarrhœa, speedily subsiding; no stool, or vomiting of solid food, for three days before death. | The liver much enlarged, and forming the epigastric tumour; its surface variegated, and raised, by large white and apparently fatty tubercles developed in its substance; its left lobe less tuberculated, but discoloured and readily lacerable. A large gall stone in the gall-bladder. The right portion of the little omentum greatly thickened, and reesmbling, in colour and consistence, the hepatic tubercles; pylorus contracted, indurated, thickened, ulcerated internally, and, externally, in the part corresponding to the litt'e omentum. The stomach filled by a brown fluid. |

| | *Sex, Age, Habits, &c. of the Subject.* | *Morbid Phænomena observed during Life.* | *Morbid Appearnces on Dissection.* |
|---|---|---|---|
| IX. | Male, aged sixty-three. Period from July to Oct. 1796. | Indigestion; pain in the epigastric region; vomiting at intervals, general dropsy, bowels torpid; epigastric pain and other symptoms aggravated; the ejected matter coloured by a sooty fluid; hiccough; increased effusion, terminating in death. | Great effusion of serum into the abdomen; the contracted pylorus, and adjacent portion of the stomach, occupied by a scirrhous tumour, of almost cartilaginous hardness, exhibiting several ulcerated points; the gastric parietes elsewhere flaccid, and readily lacerable. A plaster-like enlargement of the mesenteric glands. |
| X. | Male, aged sixty. Period from middle of May to end of June, 1797. | Epigastric pains, of a month's duration; mouth foul and bitter; disposition to vomit; head-ache: vomiting increased in force and frequency. An unnatural resistance in the epigastrium; suffering aggravated. Matter voided by vomiting and stool, blackish and viscous; no sleep; exhaustion; death. | Pylorus, with a mere fissure for its orifice, and adjacent parts of the stomach, forming a firmish lymphatic tumour partly disorganized. |

Reviewing the *ten cases* of *pyloric scirrhus,* just delineated, we observe that *eight* of them occurred in the *male,* and *two* in the *female subject;* that *one* of the patients only was *below* the *fiftieth,* and *six* of them were *beyond* the *sixtieth year;* and that mental or bodily distress had been endured by several. *Pains in the stomach,* and *vomiting* usually *some hours after* the reception of food, existed in *all* the cases. *Costiveness* is noted to have prevailed in *nine; emaciation,* in *six; dyspnœa,* in *three.* An *epigastric tumour* was externally discoverable in *seven. Five* of the cases were *uncomplicated* with other lesion. *Two* displayed *complication* with *enlarged* and *tuberculated liver; two,* with *abdominal and general dropsy; two,* with *diseased omentum; one,* with *scirrhous pancreas;* and *one,* with *enlarged mesenteric glands.*

It was our intention to have instituted, in this place, an inquiry into the practicability and means of discriminating the three varieties of gastric scirrhus, the cardiac, the central, and the pyloric, from each other; or into what may be designated the *particular,* as opposed to the *general diagnosis* of scirrhous affections of the stomach: but such discussion, we think, on reconsidering the matter, will be most advantageously introduced into our review of Dr. Chardel's section on Diagnosis; and thus, that precision in arrangement, for which we are such zealous advocates, will remain unviolated. We lament that, from Dr. Chardel's omission of the subject, the task has devolved upon a hand so much less capable of its successful execution than his own. That correct discrimination of the three varieties of gastric scirrhus now under review, is very frequently, though not invariably, practicable, we are convinced; and to those, who may declaim upon the inutility of such refinements as regards practice, we would reply, that advances in the science of diagnosis, like those in other paths of human knowledge, ought not to be spurned or rejected, because of our inability, at present, to descry all, or any, of the beneficial consequences of which they may become eventually productive.

The number of facts, presented in the three preceding sections of Dr. Chardel, however considerable in point of individual exertion, and reflecting honour on his zeal as a pathologist, is, we are well aware, too limited, to render any conclusion drawn from them perfectly legitimate and incontrovertible. Yet no mischief can result from applying to them the test of induction, and stating the results, as applicable to the *particular* diagnosis of gastric scirrhus; provided the source of fallacy, just specified, be duly recollected, and care be taken to rectify our calculation by the light of future experience. Such a retrospect will not, perhaps, be found utterly destitute of utility in the prosecution of our subject; although, it must be acknowledged, we undertake it rather in the fear of neglecting, than in the hope of eliciting, any thing calculated to throw direct light upon the obscure path of diagnosis, which, in the absence of a better guide, we shall presently lead on the young pathologist to explore.

From a cautious review of the twenty histories of scirrhus of the stomach delivered by Dr. Chardel, the following conclusions, *with respect to his sphere of observation and practice,* may evidently be drawn:

*Cardiac* and *central scirrhus* are *less* frequent than the *pyloric,* in the proportion of *one* to *two*.

*Cardiac scirrhus,* in the *female* and *male sexes,* occurs in the relative proportion of *two* to *five*; the other *two varieties,* in that of *one* to *five:* the *general proportion* being that of *five* to *twenty.*

*Age* apparently exerts some influence in determining the variety of gastric scirrhus; the mean age of the *ten victims* of *cardiac* and *central scirrhus* being 49 *and* ½ *years;* while the *ten subjects* of the *pyloric lesion* present an average of 57 *and* ½.

*Vomiting invariably* exists in *cardiac* and *pyloric scirrhus;* but is *absent* in *one* case, of *five,* of *central scirrhus.**

*Emaciation constantly* signalizes *cardiac scirrhus;* but in the *other two varieties* apparently prevails only in *three* cases out of *five.*†

*Constipation* is an attendant on *pyloric, cardiac,* and *central scirrhus,* in the respective proportions of *nine, eight,* and *six,* to *ten.*

The existence of an *evident tumour in the epigastric region* becomes progressively more frequent, as we pass from the œsophageal to the duodenal extremity of the stomach. Thus it is found bearing the respective proportions of *four, six,* and *seven,* to *ten,* in *cardiac, central,* and *pyloric scirrhus.*

Here we must for a while pause. The great length to which our analysis of the three morbid anatomical sections has already been extended, constrains us reluctantly to postpone to our next Number the examination of the doctrinal and practical portion of Dr. Chardel's most interesting monograph.

To those who may consider that we are here allotting a space in our department of Review, utterly disproportioned to the magnitude of this little volume, we beg leave to address a parting observation. Foreign works upon medicine, however celebrated or valuable, have rarely paid for the expence and trouble attendant on their translation into the English language; much less are they likely to do so in the present dark season of national calamity and distress: and such performances, when undertaken, are frequently executed by an unqualified or negligent hand. With a view, then, to compensate for the non-existence or imperfection of such translations, we shall, on all occasions, analyze important foreign publications with especial accuracy and mi-

---

* *Pain* we need not mention; for it is more or less present in *every variety* of gastric scirrhus, and hence obviously inapplicable to the *particular* diagnosis of these affections. *Dysphagia* is occasionally present in *cardiac scirrhus;* and *dyspnœa,* more frequently, in the *pyloric variety.* Rev.

† The correctness of this inference evidently depends on Dr. Chardel's fidelity of observation and detail. *Emaciation,* however, we believe, will be *invariably* found in *all* cases of scirrhus of the stomach, where life has not been destroyed by other supervening or complicated disease, ere the gastric lesion have reached its concluding stage. Rev.

nuteness; and thus offer a sketch, which, while universally accessible, from its price and language, to all orders of the profession, will be sufficiently detailed for every purpose of practical utility. How far such principles and exertions will command the respect, or be honoured by the approbation, of the public, time alone can determine. We look forward with confidence to the result. But neglect would be sweetened, or even failure disarmed of its sting, to us, by the proud consciousness which such principles, and such exertions, cannot fail to inspire.

---

